# Effects of prenatal exposure to extremely low electro-magnetic field on in vivo derived blastocysts of mice

**Published:** 2012-11

**Authors:** Parvin-Dokht Bayat, Mohmmad Reza Darabi, Ali Ghanbari, Sara Amiri, Pardis Sohouli

**Affiliations:** 1 *Department of Anatomy, Arak University of Medical Sciences, Arak, Iran.*; 2 *Fertility and Infertility Research Center, Kermanshah University of Medical Sciences, Kermanshah, Iran.*; 3 *Student Research Committee, Kermanshah University of Medical Sciences, Kermanshah, Iran.*

**Keywords:** *Blastocyst*, *Electromagnetic fields*, *Mice*

## Abstract

**Background: **Indisputable population exposure to widespread electromagnetic fields, has grown concerns over the probable health effects of these fields.

**Objective:** The present study was aimed to examine the possible effects of 50 Hz extremely low frequency electromagnetic field (ELF-EMF) exposure on the number and quality of mice blastocysts.

**Materials and Methods:** In present study, 66 NMRI pregnant females divided into two treated and non-treated groups. The treated group exposed to ELF-EMF (50 Hz and 6×10^-3^ T). Subsequently, embryos were collected by flushing the uterine horn and Fallopian tubes on the day 3 of gestation. Number of trophoectoderm (TE) and Inner Cell Mass (ICM) cells in blastocysts were determined after differential nuclei staining using a modified method. Furthermore, number of all flushed blastocysts calculated in each group.

**Results:** There was no significant difference in mean number of blastocysts in treated (6.64±1.34) and none treated (8.22±1.59) groups. In treated group, there were significant decreased in total cell number of blastocysts (p=0.000), number of ICM cells (p=0.000), and number of TE cells (p=0.001) whereas the ratio of ICM/TE cells increased (p=0.002).

**Conclusion:** The data indicate that ELF-EMF is able to affect cellular composition of blastocysts, but it can't omit total volume of blastocysts.

## Introduction

Nowadays, widespread applications of electrical instruments have resulted in population continuous exposure to electromagnetic fields (EMF), produced by electric current flows. 

The probable effects of these fields, particularly on human health, have attracted researchers' attention to perform more investigations (1). Recent investigations have manifested that even small changes of EMF intensity can induce biological disorders (2). The most prominent EMFs expose that modern societies in ordinary life are Non-ionizing 50-60 Hz ELF-EMFs (3). 

Studies, assessing the reproductive influences of power frequency (50-60 Hz) exposure, demonstrated different and sometimes adverse results in males and females. Increased risk of miscarriage caused by ELF-EMF exposure during pregnancy in women, changes in uterus and ovary in rats, congenital deformities and developmental delay in the offspring of mice, increased height of mice fallopian tube epithelial cells and reduced fertility in female rats are some outcomes of these investigations in females (4-10). It has also been investigated that 50-Hz magnetic field reduces the secretion of hormones and increases the apoptosis-related gene expression in human villous trophoblasts *in vitro *(11, 12).

Although, in recent years, many human diseases have been suspected to associate with ELF-EMF, by means of performing *in vitro* experiments, but there has not been any definitive evidence to prove these associations. Thus, ELF-EMF involvement in onset of disorders has remained somewhat controversial. Starting from these premises, present research aimed to judge the probable effects of 50 Hz ELF-EMF, on the quality and features of mice blastocysts.

## Materials and methods

**Animals**

In this experimental study, sixty six fertilized female NMRI (Naval Medical Research Institute) mice (8-12 weeks of age) were subjected to the experiments according to moral code: 5-11-6-88 of Arak University of Medical Sciences. The mice were randomly divided into 2 groups (33 animals per group): Group I (non- treated group) was not exposed to ELF-EMF and Group II (treated group) was exposed to ELF-EMF for 48 hours. Embryos were obtained by flushing the uterine horn and fallopian tubes on the day 3 of gestation with CMRL 1066 culture medium (Gibco; 21530-076) with 1milli-mol/liter L-glutamine (Sigma; G7513) and 100mM sodium pyruvate (Sigma; P8574). 

**Exposure system**

The ELF-MF used in the present study was produced by a pair of Helmholtz coils able to generate a highly homogeneous field (with homogeneity 5/1000) (13, 14). To avoid changing in heat and electromagnetic field, hose water was passed around sinusoid. The characteristics of the system were as follows: 

(I) Power supply: 220 V in, 25 V out, permanent current intensity 3 Ampere. 

(II) Multi-meter to control the intensity of the current entering the instrument. 

(III) A 50 Hz sinusoidal oscillating ELF-MF was produced by a 380 round turn coil twisted around a cylinder (19 cm diameter and 15.5 cm length) and containing a chamber to house the mice in the center of the cylinder, where the maximum even ELF-MF (6±0.1 mT) and temperature (37±0.1^o^C) was recorded.

(IV) A Teslameter (compensation-51662, sensitivity ≥0.1 mT) was used for precise measurement of magnetic field intensity in the chamber.

**Differential staining of blastocysts**

The numbers of blastocysts in two groups were counted (n=490.36±1.46). Then, twenty five blastocysts of each group were randomly selected. Trophoectoderm cells (TE) and cells of Inner Cell Mass (ICM) were counted after differential nuclei staining using a modified method of Piekos *et al* (15). Briefly, embryos were submitted to zona removal using Tyrods’ solution (pH=2.2). 

The zona-free blastocysts were incubated at 5^o^C in M16 medium (Sigma; M7292) containing 10Mx10^-3^ trinitrobenzenesulphonic acid, 4.0gx10^-3^/lx10^-3^ polyvinylpyrolidine and 0.015w/w Triton X-100 for 10 minutes. 

After washing in M2 medium (Sigma; M7167), the blastocysts were incubated in 0.1gx10^-3^/l x10^-3^ anti-dinitrophenol-BSA at 37^o^C for 15 minutes and washed again with M2 medium in triplicate. The blastocysts were then incubated in M2 medium containing a 1:10 dilution of guinea pig complement serum (EMD Chemicals; 234395) and 10 g/ml propidium iodide (Sigma; 81845) at 37^o^C for 15 min and washed in Dulbecco’s PBS (Gibco; D8537) in triplicate. 

**Figure 1:**
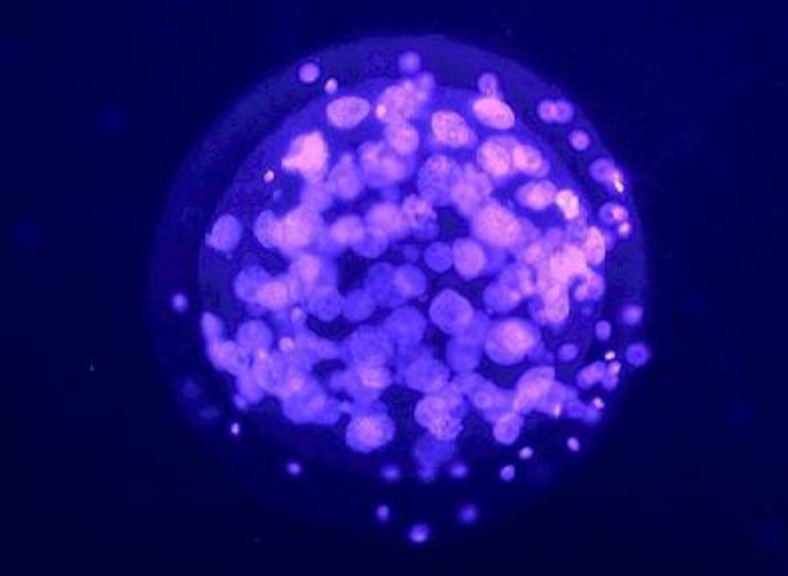
Exposed blastocysts were examined under fluorescent microscope to identify ICM cells (stained light blue) and TE cells (stained pink) (×100).

After fixing in absolute ethanol containing 22g/ml bisbenzimide (Sigma; B 2261) at 5^o^C overnight, individual blastocysts were mounted in glycerol on microscopic slides and compressed manually before visualizing by epi-fluorescence (Nikon; 801) using Nikon filters; UV-2A and G-2A. Blue nuclei were considered as originating from the inner cells (ICM) and red-to- pink fluorescing nuclei as belonging to the outer cells (TE) (Figure 1).

**Statistical analysis**

In this study all of the parameters were stated as means±SEM. The number of blastocytes, ICM and TE cells and also the ratio of ICM/TE cells were determined repeatedly four times and the standard deviations were calculated. Statistical analysis was done by paired T-test using SPSS 16.0 for Windows XP (SPSS Inc., Chicago, IL). p<0.05 was considered significant.

## Results

The mean number of blastocysts, total cells in each blastocyst, ICM cells, TE cells and also the ratio of ICM to TE cells are shown in table I. The data indicated that there was no significant difference in the numbers of blastocysts in two groups (p=0.07) (Table I). The information about the quality of obtained blastocysts from mice following maternal exposure to ELF-EMF, are showed in figure I and the determined data about quality in table I. 

In treated group, there were significantly decreased in the mean numbers of ICM cells (p=0.00), TE cells (p=0.01) and of total cells (p=0.00) whereas the ratio of ICM cells to TE cells was significantly increased (p=0.02). 

**Table I:** The effects of ELF-EMF on the blastocysts of mice

**Parameters of blastocysts **	**Non-treated**	**Treated**	**p-value**
Numbers of blastocysts (n. B)	8.22 ± 1.59	6.64 ± 1.34	0.07
Total cells (TC)	54.56 ± 11.38	41.49 ± 7.23	0.00**
Inner cell mass (ICM)	22.88 ± 6.2	15.75 ± 3.41	0.00**
Trophoectoderm cells (TE)	31.67 ± 6.05	25.74 ± 4.86	0.01**
ICM/TE	0.42 ± 0.14	0.62 ± 0.12	0.02*

The data of paired t test are shown as mean±SEM

* p<0.05

** p<0.01

## Discussion

The present study showed that exposure to ELF-EMF is not able to affect the number of blastocysts. This data is similar with Hannele *et al* that showed that *in vitro* exposure to 50 Hz magnetic field did not induce considerable difference in the number of mouse early blastocyst and late blastocysts (16). 

In parallel, Pafkova *et al* found no significant alterations in embryogenesis of rat and chick embryos, exposed to 50 Hz EMF at 6 or 10 tesla x10^-6^ of intensity. These results are in contrast with Borhani *et al* that indicated significant decrease in the mean number of mice blastocysts at the result of exposure to 50-Hz EMF. This controversy could be due to the duration of exposure that was 48 hours in this study while Borhani *et al* exposed pregnant mice for 12 days (17, 18).

However, the study showed that numbers of ICM cells, TE cells and total cells in blastocysts of ELF-EMF exposed group were significantly decreased. These indisputable decreases in mentioned numbers, indicates growth depression inducement in ELF-EMF exposed group. 

Thus, present study confirmed the cellular toxicity of prenatal exposure to ELF-EMF as the same as that for adult rats (7). Although the mechanisms by which ELF-MF induce cytototoxicity is not completely understood, apoptosis is considered as main cellular event by many authors. *In vivo* studies showed that 50 Hz 0.2, 3.2 or 6.4 mT for 2 weeks or 4 weeks blocked the cell cycle in S phase in testes of mice and 60 Hz 14 mT magnetic field for 8 weeks induced apoptosis in testicular germ cell in mice (19, 20). 

*In vitro* studies confirmed in vivo experiments that showed induction of apoptosis in K562 human leukemia cell line by ELF-MF (1 mT, 50 Hz), in isolated human osteoclasts by pulsed electromagnetic fields (7.5 Hz), in isolated liver cells and peripheral blood sampled from newborn mice by ELF (50 HZ), in human normal and cancer cells by time-varying magnetic fields (60-Hz 6 mT), and also induction of micronuclei in rat bone marrow by (910-MHz) (21-25). In sub cellular level, regulations of many molecules were considered for induction of apoptosis by ELF-MF. The studies suggest formation of free radicals such as reactive oxygen species (ROS), heat-shock protein 70 (hsp70), cyclic adenosine mono phosphate (cAMP) and also P53 gene and its related gene; P38 and other regulating genes of apoptosis such as bcl-2 and bax (21, 24, 26-30).

In conclusion, this study indicated that exposure to ELF-EMF influence female mice fertility by affecting cellular composition of blastocysts. Equal number of blastocysts in treated and non-treated indicated that cytotoxic mechanisms such as apoptosis could not be enough to omit total volume of blastocysts. 

## References

[1] Salzinger K (1994). Behavioral effects of electromagnetic fields in animals: Biological effects of electric and magnetic fields.

[2] Schuz J, Mann S (2000). A discussion of potential exposure metrics for use in epidemiological studies on human exposure to radio waves from mobile phone base stations. J Expo Anal Env Epid.

[3] Wood AW (2006). How dangerous is mobile phones, transmission masts, and electricity pylons?. Arch Dis Child 2006.

[4] Li DK, Odouli R, Wi S, Janevic T, Golditch I, Bracken TD (2002). A population based prospective cohort study of personal exposure to magnetic fields during pregnancy and the risk of miscarriage. Epidemiology.

[5] Vaughn TL, Daling JR, Starzyk PM (1984). Fetal death and maternal occupation: an analysis of birth records in the State of Washington. J Occup Med.

[6] Aksen F, Akdag MZ, Ketani A, Yokus B, Kaya A, Dasdag S (2006). Effects of 50-Hz 1-mT magnetic field on the uterus and ovaries of rats (Electron microscopy evaluation). Med Sci Monit.

[7] Al-Akhras MA (2008). Influence of 50 Hz magnetic field on sex hormones and body, uterine, and ovarian weights of adult female rats. Electromagn Biol Med.

[8] Cao YN, Zhang Y, Liu Y (2006). Effects of exposure to extremely low frequency electromagnetic fields on reproduction of female mice and development of offspring. Zhonghua Lao Dong Wei Sheng Zhi Ye Bing Za Zhi.

[9] Rajaei F, Borhani N, Sabbagh-Ziarani F, Mashayekhi F (2010). Effects of extremely low-frequency electromagnetic field on fertility and heights of epithelial cells in pre-implantation stage endometrium and fallopian tube in in mice. ChinJ Integr Med.

[10] Al-Akhras MA, Elbetieha A, Hasan MK, Al-Omari I, Darmani H, Albiss B (2001). Effects of extremely low frequency magnetic field on fertility of adult male and female rats. Bioelectromagnetics.

[11] Sun W, Tan Q, Pan Y, Fu Y, Zhang D, Lu D (2010). Superimposition of an incoherent magnetic field eliminated the inhibition of hormone secretion induced by a 50-Hz magnetic field in human villous trophoblasts in vitro. Cell Physiol Biochem.

[12] Sun W, Tan Q, Pan Y, Fu Y, Sun H, Chiang H (2010). Effects of 50-Hz magnetic field exposure on hormone secretion and apoptosis-related gene expression in human first trimester villous trophoblasts in vitro. Bioelectromagnetics.

[13] Oztas B, Kalkan T, Tuncel H (2004). Influence of 50 Hz frequency sinusoidal magnetic Field on the blood brain barrier permeability of diabetic rats. Bioelectromagnetics.

[14] Bayat PD, Ghanbari A, Saeid B, Khazaei M, Ghorbani R, Ayubian M (2011). Effect of exposure to extremely low electro-magnetic field during prenatal period on mice spleen. Ind J Exp Biol.

[15] Piekos MW, Frasor J, Mack S, Binor Z, Soltes B, Molo MW (1995). Evaluation of co-culture and alternative culture systems for promoting in vitro development of mouse embryos. Hum Reprod.

[16] Hannele H, Jukka JA, Hannu K (2001). Development of preimplantation mouse embryos after exposure to a 50 Hz magnetic field in vitro. Toxicol Lett.

[17] Pafkova’ H, Jera’bek J, Tejnorov’a I, Bedna’r V (1996). Developmental effects of magnetic field (50 Hz) in combination with ionizing radiation and chemical teratogens. Toxicol Lett.

[18] Borhani N, Rajaei F, Salehi Z, Javadi A (2011). Analysis of DNA fragmentation in mouse embryos exposed to an extremely low-frequency electromagnetic field. Electromagn Biol Med.

[19] Hong R, Liu Y, Yu YM, Hu K, Weng EQ (2003). Effects of extremely low frequency electromagnetic fields on male reproduction in mice. Zhonghua Lao Dong Wei Sheng Zhi Ye Bing Za Zhi.

[20] Kim YW, Kim HS, Lee JS, Kim YJ, Lee SK, Seo JN (2009). Effects of 60 Hz 14 mT magnetic field on the apoptosis of testicular germ cell in mice. Bioelectromagnetics.

[21] Garip AI, Akan Z (2010). Effect of ELF-EMF on number of apoptotic cells; correlation with reactive oxygen species and HSP. Acta Biol Hung.

[22] Chang K, Chang WH, Tsai MT, Shih C (2006). Pulsed electromagnetic fields accelerate apoptotic rate in osteoclasts. Connect Tissue Res.

[23] Udroiu I, Cristaldi M, Ieradi LA, Bedini A, Giuliani L, Tanzarella C (2006). Clastogenicity and aneuploidy in newborn and adult mice exposed to 50 Hz magnetic fields. Int J Radiat Biol.

[24] Kim J, Ha CS, Lee HJ, Song K (2010). Repetitive exposure to a 60-Hz time-varying magnetic field induces DNA double-strand breaks and apoptosis in human cells. Biochem Biophys Res Commun.

[25] Demsia G, Vlastos D, Matthopoulos DP (2004). Effect of 910-MHz electromagnetic field on rat bone marrow. Scient World J.

[26] Wu H, Ren K, Zhao W, Baojian GE, Peng S (2005). Effect of electromagnetic fields on proliferation and differentiation of cultured mouse bone marrow mesenchymal stem cells. J Huazhong Univ Sci Technolog Med Sci.

[27] Simkó M, Droste S, Kriehuber R, Weiss DG (2001). Stimulation of phagocytosis and free radical production in murine macrophages by 50 Hz electromagnetic fields. Eur J Cell Biol.

[28] Tenuzzo B, Vergallo C, Dini L (2009). Effect of 6 mT static magnetic field on the bcl-2, bax, p53 and hsp70 expression in freshly isolated and in vitro aged human lymphocytes. Tissue Cell.

[29] Hasegawa M, Zhang Y, Niibe H, Terry NHA, Meistrich ML (1998). Resistance of differentiating spermatogonia to radiation induced apoptosis and loss in p53-deficient mice. Radiat Res.

[30] Akdag MZ, Dasdag S, Aksen F, Isik B, Yilmaz F (2006). Effect of ELF magnetic fields on lipid peroxidation, sperm count, p53, and trace elements. Med Sci Monit.

